# Overexpression of bHLH domain of HIF-1 failed to inhibit the HIF-1 transcriptional activity in hypoxia

**DOI:** 10.1186/s40659-020-00293-4

**Published:** 2020-06-05

**Authors:** Fatemeh Sadeghi, Gholam Ali Kardar, Mohammad Reza Bolouri, Farzad Nasri, Maryam Sadri, Reza Falak

**Affiliations:** 1 Immunology Research Center, Iran University of Medical Sciences, Tehran, Iran; 2 Department of Immunology, School of Medicine, Iran University of Medical Sciences, Tehran, Iran; 3 Immunology Asthma & Allergy Research Institute, Children’s Medical Center, Tehran University of Medical Sciences, Tehran, Iran

**Keywords:** Cancer, Hypoxia, HIF-1, Arnt, bHLH, PAS, Gene therapy, Hypoxia inducible factor, Basic helix-loop helix, DNA binding

## Abstract

**Background:**

Hypoxia inducible factor-1 (HIF-1) is considered as the most activated transcriptional factor in response to low oxygen level or hypoxia. HIF-1 binds the hypoxia response element (HRE) sequence in the promoter of different genes, mainly through the bHLH domain and activates the transcription of genes, especially those involved in angiogenesis and EMT. Considering the critical role of bHLH in binding HIF-1 to the HRE sequence, we hypothesized that bHLH could be a promising candidate to be targeted in hypoxia condition.

**Methods:**

We inserted an inhibitory bHLH (ibHLH) domain in a pIRES2-EGFP vector and transfected HEK293T cells with either the control vector or the designed construct. The ibHLH domain consisted of bHLH domains of both HIF-1a and Arnt, capable of competing with HIF-1 in binding to HRE sequences. The transfected cells were then treated with 200 µM of cobalt chloride (CoCl_2_) for 48 h to induce hypoxia. Real-time PCR and western blot were performed to evaluate the effect of ibHLH on the genes and proteins involved in angiogenesis and EMT.

**Results:**

Hypoxia was successfully induced in the HEK293T cell line as the gene expression of VEGF, vimentin, and β-catenin were significantly increased after treatment of untransfected HEK293T cells with 200 µM CoCl_2_. The gene expression of VEGF, vimentin, and β-catenin and protein level of β-catenin were significantly decreased in the cells transfected with either control or ibHLH vectors in hypoxia. However, ibHLH failed to be effective on these genes and the protein level of β-catenin, when compared to the control vector. We also observed that overexpression of ibHLH had more inhibitory effect on gene and protein expression of N-cadherin compared to the control vector. However, it was not statistically significant.

**Conclusion:**

bHLH has been reported to be an important domain involved in the DNA binding activity of HIF. However, we found that targeting this domain is not sufficient to inhibit the endogenous HIF-1 transcriptional activity. Further studies about the function of critical domains of HIF-1 are necessary for developing a specific HIF-1 inhibitor.

## Background

In the past two decades, gene-therapy has received great attention in the fields of medical sciences. Although it could be more favorable for the treatment of highly prevalent diseases such as cancer, the most effective results have been reported from monogenic diseases, as they are caused by a mutation in a single gene [1]. Cancer is one of the most complicated diseases for achieving a successful gene therapy. So that, in spite of several ongoing clinical trials in different countries, only a few of them show promising benefits, indicating that cancer gene therapy requires further modifications to be applied in routine clinical practice [1, 2]. To achieve a successful gene therapy, first of all, we should have a clear picture of the genetic basis of the studied disease. Furthermore, identification of the most prominent key proteins and understanding the functions and interactions of the involved protein domains is necessary. Finally, the overlapping of different signaling pathways should be clearly considered [1, 3].

Hypoxia is commonly observed in the local microenvironment of solid tumors and plays a critical role in cancer progression [4, 5], metastasis [6], therapeutic resistance [7–9], and poor prognosis [6], making it an attractive target for cancer therapy. In contrast to normal cells, cancer cells activate several pathways at decreased oxygen tensions to survive and to continue their development. Hypoxia inducible factor-1 (HIF-1) is the most prominent transcriptional factor induced by cancer cells in possibility leading to an aggressive phenotype following hypoxic conditions [10, 11]. HIF-1 has a heterodimeric structure and consists of two basic helix-loop-helix (bHLH) proteins, including HIF-1a and aryl hydrocarbon receptor nuclear translocator (Arnt). In contrast to Arnt, which has constitutive expression, HIF-1a has a short half-life and degrades following hydroxylation and ubiquitination [12, 13]. Hypoxia, oncogenes, and chemical reagents such as cobalt may stabilize HIF-1a and cause accumulation of this protein in the cells [14]. In these conditions, HIF-1a migrates into the nucleus, dimerizes with Arnt, and binds the hypoxia element response (HRE) sequence in the promoter of related genes like vascular endothelial growth factor (VEGF), N-cadherin, and vimentin [15, 16]. In addition to HIF-1a, Arnt also dimers with dioxin receptor (DR) [17] and binds to the xenobiotic response element (XRE) in the promoter of target genes such as cytochrome P4501A1 (CYP1A1) in order to promote the transcription of xenobiotic-metabolizing enzymes [18].

Considering the critical role of HIF in different steps of cancer progression, several attempts have been done to target this pathway. Based on personalized medicine, a specific HIF-1 inhibitor could be applied for patients with high HIF activity. This type of patient stratification may also help to avoid unnecessary therapies and result in a more favorable prognosis [10]. However, HIF is a complex pathway, overlapping mechanisms and signaling cascades, therefore, developing a HIF specific inhibitor is a major challenging task. Although some agents can indirectly affect the HIF-1 pathway [10]; up to now, no specific HIF-1 inhibitor has been clinically approved. For example, echinomycin, a natural cyclic peptide belonging to the quinoxaline antibiotic family, has been suggested as a small inhibitor that interferes with the HIF-1–DNA interaction [19]. However, echinomycin is not the HIF specific inhibitor and affects other transcription factors that have similar core-binding sites in their DNA consensus sequences [19]. Moreover, confirmation of its cytotoxicity in several clinical trial phases has limited its clinical application [10]. Nonspecific inhibitors are usually associated with adverse side effects and limited therapeutic efficacy. It is thus necessary to develop novel and more specific inhibitory molecules, capable to hinder HIF-1 pathway while escaping other pathways. Deciphering the molecular structure of HIF-1a domains and their critical functions in this pathway will help to design novel strategies for further improving the efficacy of current drugs and possibly identification of novel inhibitors [10].

Previous studies have reported that bHLH domains have a critical role in binding HIF-1a-Arnt to the HRE sequence or DR-Arnt to XRE sequence. X-ray crystallographic and nuclear magnetic resonance (NMR) studies have demonstrated that bHLH domain contains arginine and lysine residues, capable of binding the core nucleotides of HRE sequences [20, 21]. This domain inserts alpha helices into the major groove face of the HRE sequences in a pseudo-symmetric fashion, mainly through hydrogen bonds and van der Waals interactions [22].

In this regard, targeting DNA binding activity through the bHLH domain was considered as the main goal of some studies. Kong et al. indicated that the HIF-1a bHLH-PAS and Arnt bHLH-PAS truncated proteins, mediated the DNA-binding activity of HIF-1 [19]. In another study, Pongratz et al. reported that the bHLH regions of DR and Arnt molecules are sufficient for XRE recognition [23], suggesting that targeting only bHLH domain might be a promising approach. However, few studies proposed that the per-Arnt-Sim (PAS) domain also contributes to the DNA-binding activity of HIF-1 [22, 24]. PAS is mainly responsible for the dimerization of HIF-1a-Arnt and DR-Arnt. However, when PAS domain is deleted or any distinct point mutations are applied within this region, bHLH takes the PAS role in dimerization. In this case, the DNA-binding activity of this domain is reduced, while no significant disruption occurs in dimerization [24, 25].

According to the previously mentioned findings, we assumed that a truncated protein, containing the bHLH domains of HIF-1a dimerized with bHLH of Arnt, could reverse the DNA binding activity of HIF in the absence of the PAS domain and compete with the cell HIF-1 molecule in binding to the HRE sequence of downstream genes. Therefore, we designed a small inhibitory bHLH domain (ibHLH) protein, composed of both regions of HIF-1a bHLH and Arnt bHLH domains, to figure out whether it is capable to bind the HRE sequence and inhibit the activation of downstream genes in the hypoxia condition.

## Methods

### Cell culture

HEK293T cells were purchased from the Stem Cell Technology Research Center of Iran and propagated using complete medium composed of high glucose Dulbecco’s modified eagle medium (DMEM), supplemented with 10% fetal bovine serum (FBS) and penicillin–streptomycin and maintained at 37 °C in a humidified atmosphere containing 5% CO_2_.

### MTT cell proliferation assay

MTT (3-(4,5-Dimethylthiazol-2-yl)-2,5-diphenyltetrazolium bromide) cell proliferation assay was performed to assess the toxic effect of Cobalt (II) chloride (CoCl_2_) (Thermo Fisher Scientific, Miami, Florida, USA) as it is an hypoxia-mimicking substance, on the viability of HEK293T cells. Briefly, 2.5 × 10^3^ cells were cultured in complete medium in 96-well plates and during the following day were treated with 150 or 200 µM CoCl_2_ for 24–48 h. At the end of the incubation time, the media of each well was removed and replaced with fresh serum free medium, supplemented with 20 µl of MTT solution (5 mg/ml; Sigma, St. Louis, Missouri, USA). After 3 h, the MTT solution was removed and 100 µl dimethyl sulfoxide (DMSO) was added to each well and the plate was maintained at 37 °C on an orbital shaker. After 15 min, the absorbance was recorded at 570 nm using a microplate reader.

### Hypoxia induction

To induce hypoxia, 1.5 × 10^5^ HEK293T cells were treated with 150 or 200 µM concentrations of CoCl_2_ for 24–48 h. The efficiency of the hypoxia was evaluated by real-time PCR to determine VEGF gene expression and also by western blot analysis of HIF-1a at the protein level.

### Real-time PCR

Total RNA was extracted using TRIzol^®^ reagent (GeneAll, Seoul, South Korea) and qualified with NanoDrop 2000c spectrophotometer (A260/A280 ratio was 1.8–2.0 and A260/A230 ratio was > 1.8). cDNA was synthesized from 1 μg/μl of RNA according to the manufacturer’s instructions (Thermo Fisher Scientific, Miami, Florida, USA). The real-time PCR was performed in 20 μl reaction volumes, containing 10 μl of 2X SYBR-Green mix (Biofact, Daejeon, South Korea), 1 μl cDNA and 0.5–1 μl of both reverse and forward primers. Two-step thermal cycling consisted of 1 cycle of 95 °C for 5 min, followed by 40 cycles of 95 °C for 15 s and 56–60 °C, for 40 s. The details of the primers sequence and their annealing temperature are presented in Table 1. HPRT1 was considered as the housekeeping gene and 2^−(∆∆CT)^ value was calculated to get the relative expression.

**Table 1:** The details of primers sequence and the annealing temperature

Primer	Sequence (forward, reverse)	Annealing temperature
HPRT1	F: AATTACTTTTATGTCCCCTGTTGACTGG R: GCTATAAATTCTTTGCTGACCTGCTG	63
VEGF	F: TTGCCTTGCTGCTCTACCTCCA R: GATGGCAGTAGCTGCGCTGATA	62.5
HIF-1a	F: TGAGTTCGCATCTTGATAAGGC R: ACAAAACCATCCAAGGCTTTCA	63
N-cadherin	F: GGACAGTTCCTGAGGGATCA R: GGATTGCCTTCCATGTCTGT	62.5
Vimentin	F: GGTGGACCAGCTAACCAACGA R: TCAAGGTCAAGACGTGCCAGA	62.5
β-catenin	F: AAAATGGCAGTGCGTTTAG R: TTTGAAGGCAGTCTGTCGTA	62

### Western blotting analysis

HEK293T cells were washed with phosphate buffered saline (PBS) and lysed with radio immunoprecipitation assay (RIPA; Santa Cruz Biotechnology, Dallas, Texas, USA) buffer, containing protease inhibitor cocktail. Equal concentrations of whole cell lysate (approximately 60 µg), were loaded on 12.5% SDS-PAGE slabs after measurement of the protein content of the extracts with bicinchoninic acid (BCA) kit. The resolved proteins were transferred to the polyvinylidene difluoride (PVDF) membrane and the membrane was blocked with 5% skim milk in PBS containing 0.2% tween, overnight (4 °C). The membrane was incubated with mouse anti human HIF-1a (1:100, Santa Cruz Biotechnology, Dallas, Texas,), rabbit anti human N-cadherin (1/500, Abcam, Cambridge, UK), β-catenin (1/3000, Abcam, Cambridge, UK) and β–actin (1:1000, Abcam, Cambridge, UK) diluted in 2% skim milk/PBST at 4 °C overnight, followed by 3 washes in PBST (3 times × 5 min). Then, the membrane was incubated with appropriate dilutions of secondary antibodies at RT for 1 h, followed by 4 washes with PBST and final visualization using enhanced chemiluminescence reagent (Intron Biotechnology, Seongnam-Si, South Korea).

### Plasmid constructs

**Fig. 1:**
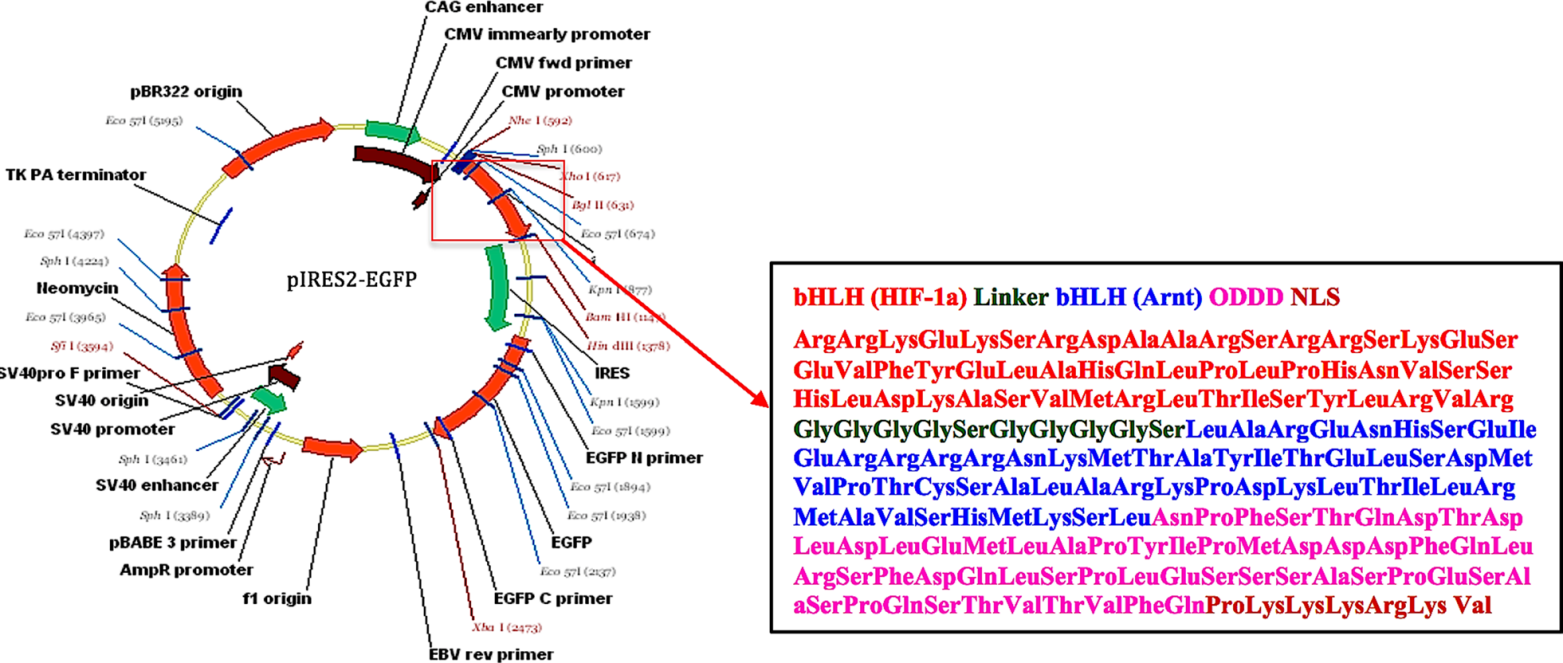
Restriction map of pIRES2-EGFP vector and the details of amino acids sequence of ibHLH protein, inserted in the multiple cloning site of pIRES2-EGFP. *bHLH* basic helix-loop helix, *HIF-1a* hypoxia inducible factor-1a, *Arnt* aryl hydrocarbon receptor nuclear translocator, *ODD* oxygen-dependent degradation, *NLS* nuclear localization signal

The ibHLH (HIF-1a bHLH domain: amino acids # 17–70, Arnt bHLH domain: amino acids # 89–142) was cloned into the multiple cloning site (MCS) of pIRES2-EGFP plasmid (Fig. 1) with a linker between them and verified by sequencing (Additional file 1). Moreover, to increase the specificity of ibHLH in hypoxia, and avoid the unwanted overexpression in normal condition, we fused the oxygen depended domain (ODD) domain to C-terminal of ibHLH (Fig. 1). ODD domain is responsible for stabilizing HIF-1 in hypoxia and causes HIF-1 degradation in normoxic conditions through ubiquitination and proteasomal degradation.

### Transient transfection

HEK293T cells were transiently transfected with either control pIRES2-EGFP or ibHLH- pIRES2-EGFP plasmids using polyethylenimine (PEI; Sigma, St. Louis, Missouri, USA) reagent. Briefly, 1.3 × 10^5^ cells were seeded in 2 ml 10% FBS medium in 6-well plates overnight. On the day of transfection, the medium was replaced with 1.5 ml of antibiotic-free medium, containing 1% FBS and incubated for 2 h. The transfection complex was prepared by adding 500 µl of serum-free and antibiotic-free medium, 5 µg/µL DNA (Control or ibHLH vectors), and 12.5 µl PEI with the respective order and incubated at RT for 10 min. The transfection complex was added dropwise to the cells. After 4 h, the medium was aspirated and replaced with complete medium. Transfection efficiency was controlled with invert fluorescent microscopy and flow cytometry methods and the side-effects of transfection on the viability of the cells were evaluated by propidium iodide staining. After 24 h of the transfection, the cells were treated with 200 µM CoCl_2_ for 48 h (as optimal concentration and time for hypoxia induction) and then were collected for molecular analysis.

### Statistical analysis

Statistical parameters and tests are reported in the legends of figures. All gene level data were presented as mean (± SD). One-way ANOVA, Bonferroni analysis was performed for all the datasets that required comparison among more than two independent groups. At the protein level, we performed nonparametric tests. The data were presented as the median (± IQR) and Kruskal–Wallis, Dunn test was performed to compare between two or more independent groups. Moreover, Benjamini–Hochberg–was done to control the False Discovery Rate (FDR) in multiple testing experiments. All of the data is presented by GraphPad Prism 7 (GraphPad Prism Software, San Diego, CA, USA) and the statistical analysis was performed using STATA/SE version 12.0 software (STATA Corp., TX, USA).

## Results

### Toxicity of CoCl_2_ on the HEK293T cells

**Fig. 2:**
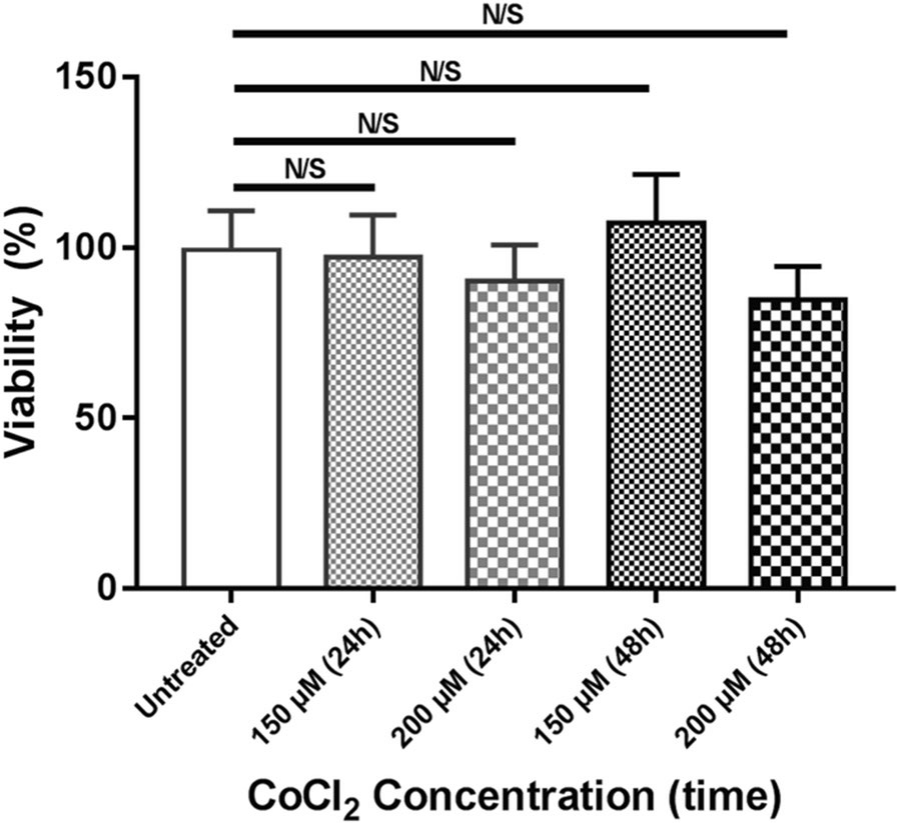
The effect of different concentration of CoCl_2_ on the viability of HEK293T after 24 h and 48 h. Data represents the mean (± SD) of the percentage of viability from two independent experiments, each performed in triplicate. Statistically analysis was performed on the percentage of viability, using One-way ANOVA, Bonferroni. Error bars indicate ± SD. (*p < 0.05, **p < 0.01, ***p < 0.001, N/S: Not significant)

The MTT assay demonstrated that both of the studied concentrations of CoCl_2_ (150 µM and 200 µM) had no significant side-effect on the viability of HEK293T cells after 24 and 48 h compared to the control group (p > 0.05) (Fig. 2).

### The induction of hypoxia with CoCl_2_

**Fig. 3:**
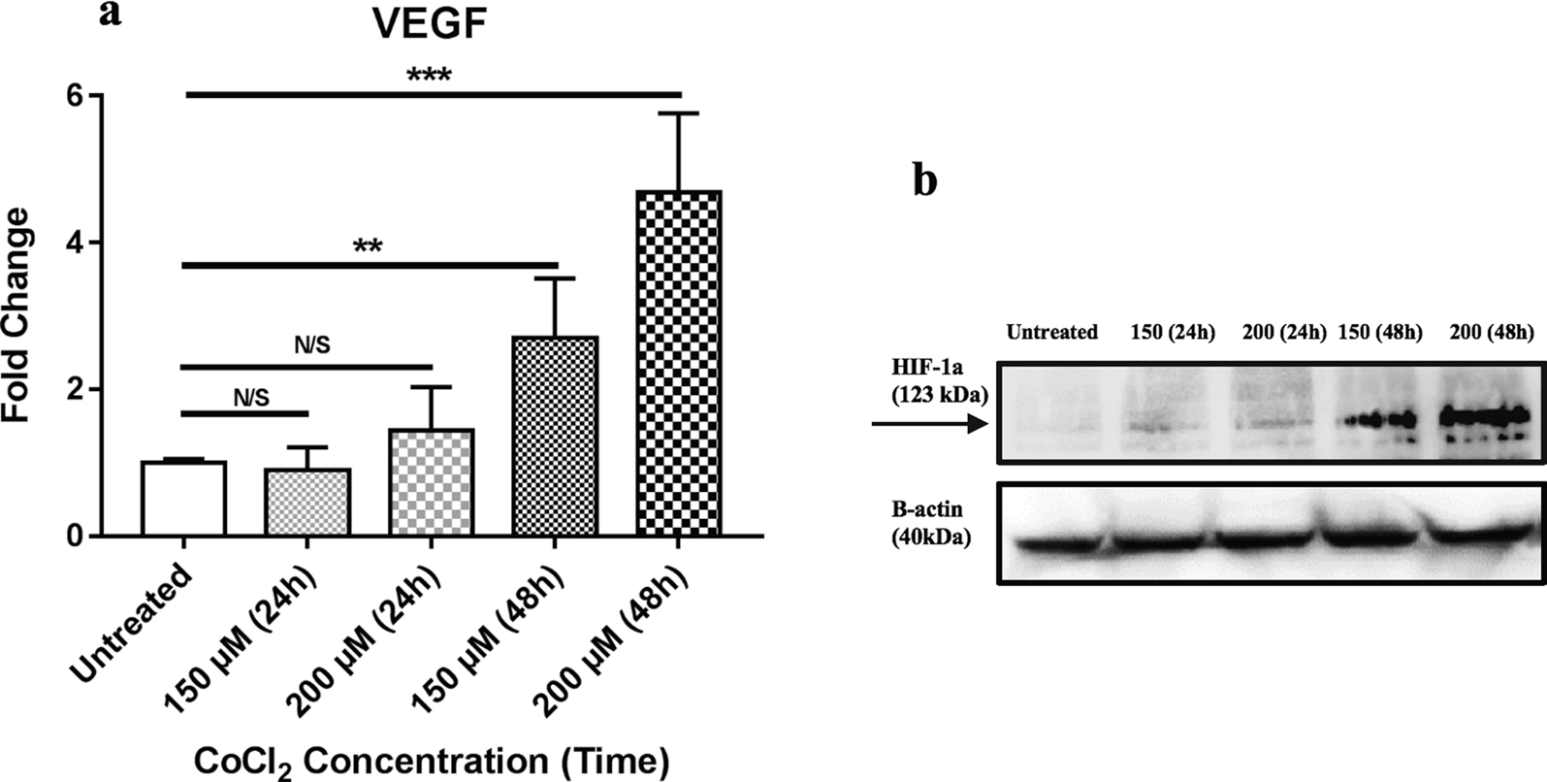
Hypoxia induction on HEK293T with CoCl_2_. **a** Fold change expression of VEGF mRNA after treating with different concentrations of CoCl_2_ for 24 h and 48 h. Data represents the mean (± SD) of fold changes from two independent experiments, each performed in triplicate. Statistically analysis was performed on Log2 Fold Change, using One-way ANOVA, Bonferroni. Error bars indicate ± SD. (*p < 0.05, **p < 0.01, ***p < 0.001, *N/S* not significant). **b** Western blot analysis of protein expression of HIF-1a after treating with different concentrations of CoCl_2_ for 24 h and 48 h

Treating HEK293T with CoCl_2_ significantly increased the expression of VEGF as the main downstream gene of HIF-1a, at both 150 µM (p < 0.01) and 200 µM (p < 0.001) concentrations after 48 h (Fig. 3a). CoCl_2_ at concentration of 200 µM induced a 4.6-fold increase on the expression of the VEGF gene. Similarly, the cellular level of the HIF-1a protein was increased in time and dose-dependent manner by CoCl_2_ (Fig. 3b). Therefore, treatment of the cells at 200 µM concentration for 48 h was considered as the optimal hypoxia condition for HEK293T cells.

### The efficiency of transfection and the viability of HEK293T cell after transfection

**Fig. 4:**
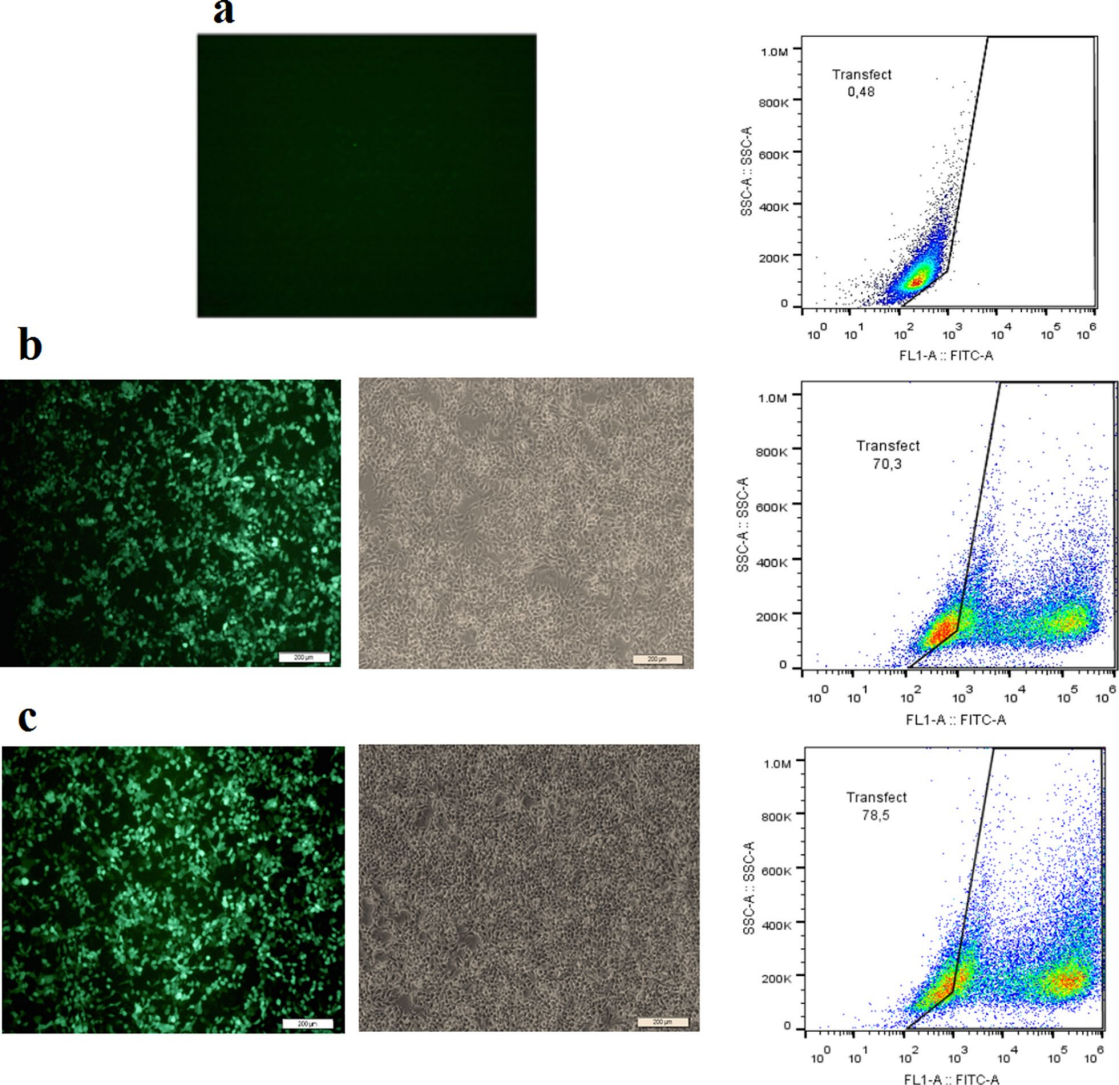
Photograph of the EGFP-transfected HEK293T obtained with a fluorescence microscope and rate of transfection obtained with flow-cytometry, 48 h after transfection. **a** Un-transfected cells, **b** Transfected with control pIRES2-EGFP, and **c** Transfected with ibHLH pIRES2-EGFP

**Fig. 5:**
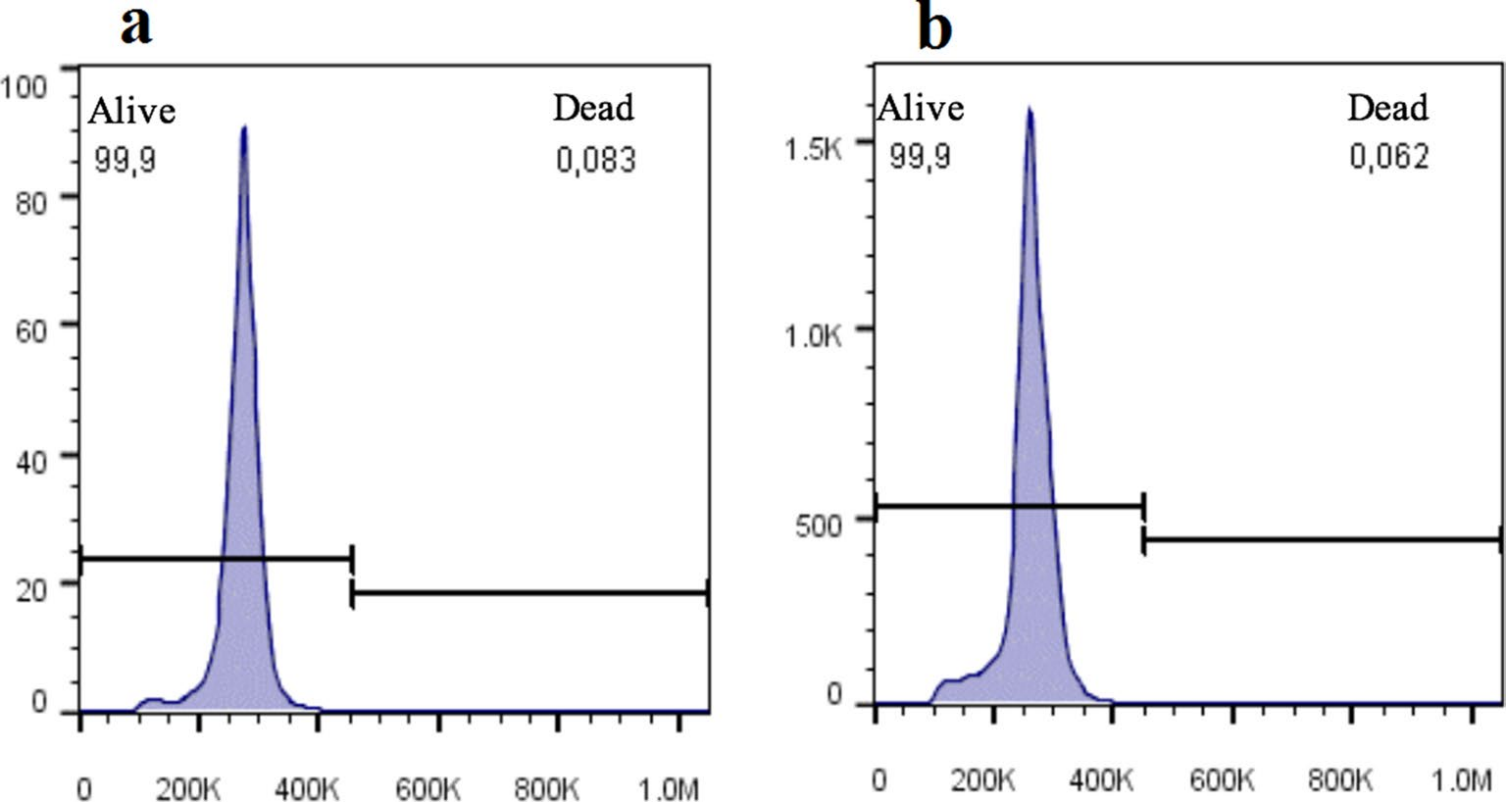
The effect of transfection on the viability of HEK293T, 48 h after transfection by Propidium Iodide staining. **a** Un-transfected HEK293T cells, and **b** Transfected with ibHLH pIRES2-EGFP

The rate of transfection of HEK293T with control pIRES2-EGFP and ibHLH pIRES2-EGFP were 78% and 87% respectively, 48 h post-transfection (Fig. 4) and the transfection assay had no significant effect on the viability of HEK293T cells (Fig. 5).

### The effect of ibHLH on the expression of different genes, involved in angiogenesis and EMT of tumor cells

**Fig. 6:**
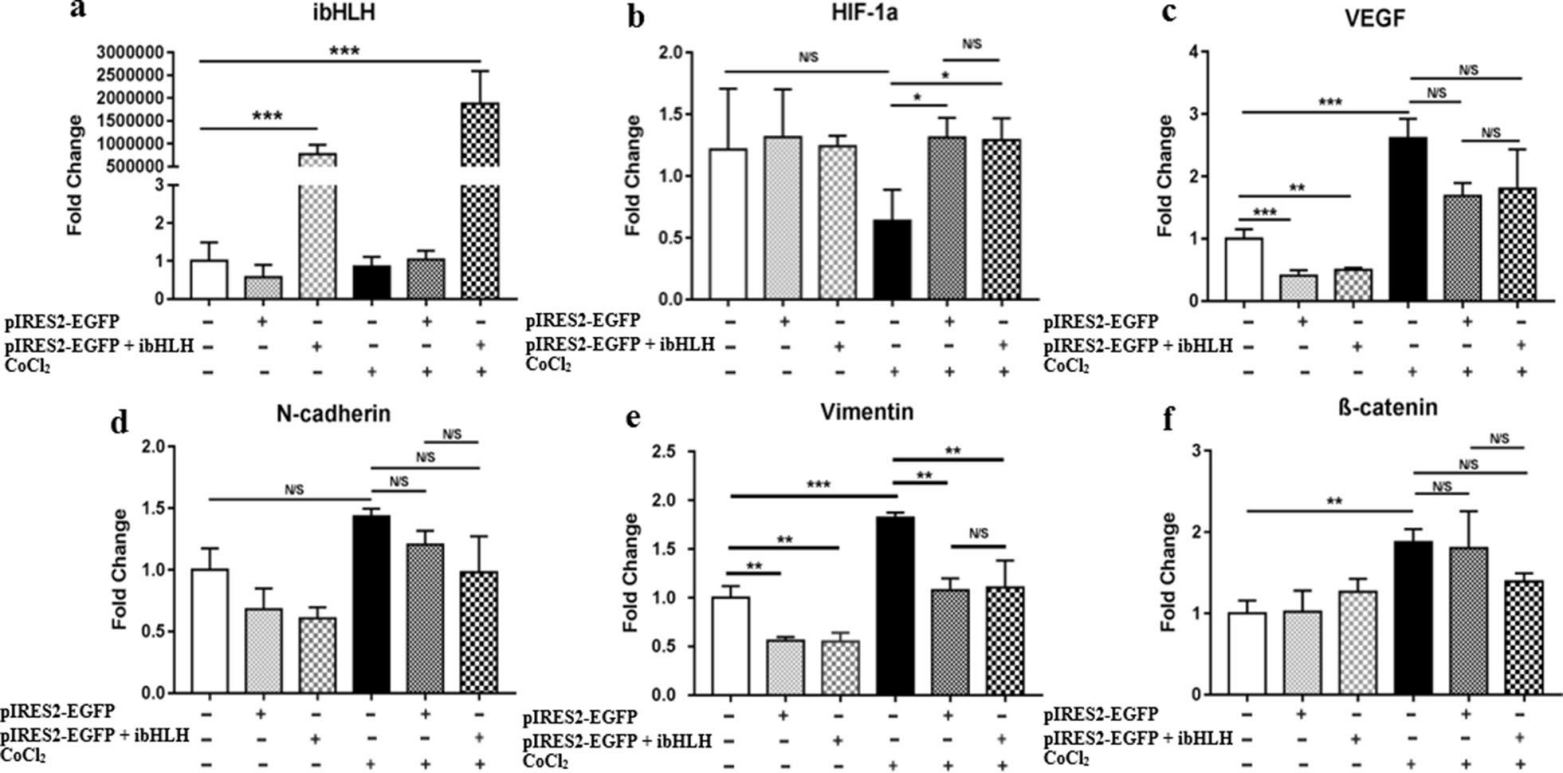
The effect of ibHLH pIRES2-EGFP on the hypoxic-depended expression of ibHLH (**a**), HIF-1a (**b**), VEGF (**c**), N-cadherin (**d**), Vimentin (**e**), and β-catenin (**f**). Data represent the mean (± SD) of Fold Changes from two independent experiments, each performed in duplicate. Statistically analysis was performed on Log2 Fold Changes, using One-way ANOVA, Bonferroni. Error bars indicate ± SD. (*p < 0.05, **p < 0.01, ***p < 0.001, *N/S* not significant)

As expected, gene expression of VEGF (Fig. 6c, p < 0.001), vimentin (Fig. 6e, p < 0.001), and β-catenin (Fig. 6f, p < 0.01) were significantly increased after treatment of untransfected HEK293T cells with 200 µM CoCl_2_ for 48 h.

Gene expression of VEGF, N-cadherin, vimentin and β-catenin was decreased in the cells transfected with either control or ibHLH vectors during hypoxia. However, this reduction was only statistically significant for the case of vimentin (p < 0.01).

Regarding the net effect of ibHLH, we observed that ibHLH had more inhibitory effects on gene expression of N-cadherin and β-catenin compared to the control vector. However, it was not statistically significant. Moreover, there was no difference in hypoxia-depended expression of VEGF and vimentin between the cells transfected with ibHLH and control vector.

### The effect of ibHLH on the protein expression of N-cadherin and β-catenin

**Fig. 7:**
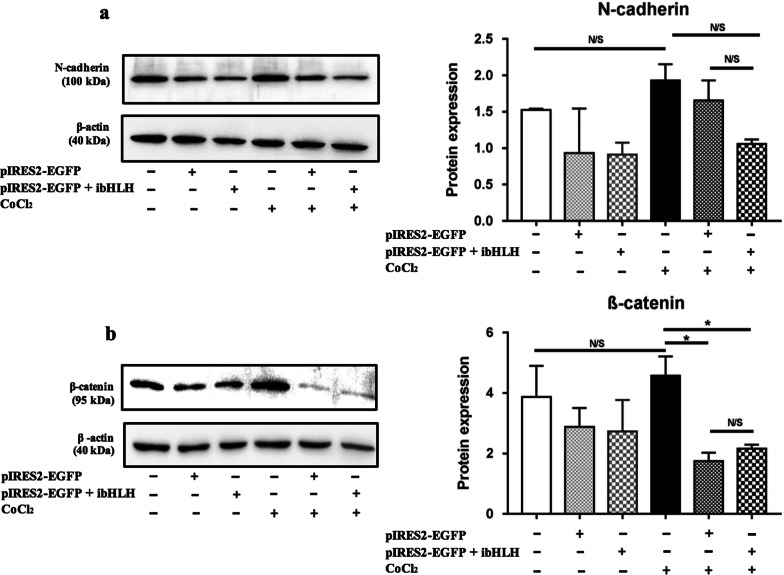
The effect of ibHLH pIRES2-EGFP on the protein expression of N-cadherin (**a**), and β-catenin (**b**). Data represent the median (± IQR) of normalized protein expression with β-actin from one experiment, performed in triplicate. Statistically analysis was performed on protein expression, using Kruskal–Wallis, Dunn test, Benjamini–Hochberg. Error bars indicate ± IQR. (*p < 0.05, **p < 0.01, ***p < 0.001, *N/S* not significant)

Similarity to what occurred at the gene level, at the protein level, we observed that ibHLH reduced protein expression of N-cadherin more than the control vector (Fig. 7a). However, it was not statistically significant (p > 0.05). Moreover, we could not find any difference between the control and the ibHLH vectors in reducing the protein level of β-catenin (Fig. 7b).

## Discussion

HIF-1 is the most prominent transcriptional factor of hypoxia and has crucial involvement in different hallmarks of cancer phenotypes, making it an attractive target for developing a novel therapeutic approach in the field of personalized medicine. In this study, we developed a new specific HIF-1 inhibitor protein by targeting the bHLH domains of this transcriptional factor (ibHLH), in order to inhibit the DNA binding activity of HIF-1. We found that both the control and the ibHLH vectors decreased the hypoxia-depended gene expression of VEGF, N-cadherin, vimentin, and β-catenin as well as the protein level of β-catenin. However, there was no significant difference between the control vector and the ibHLH vectors in reducing the gene and their protein levels. It seems that the overexpression of ibHLH protein is not sufficient to significantly inhibit endogenous HIF-1 transcriptional activity and more studies about the exact molecular structure of the domains that mediate critical functions of HIF-1 is necessary for developing a specific HIF-1 inhibitor.

Hypoxia as a prominent feature of solid tumors has been targeted in several studies. However, most of the HIF-1 inhibitors described so far in the literature are non-specific; thus, differentiation of their contribution to HIF-1 and other pathways is difficult. Therefore, development of further specific inhibitors that could selectively inhibit HIF-1; needs to be studied.

The critical role of bHLH in DNA binding has been reported in several studies [20–22, 26]. HIF-1a-Arnt recognizes the HRE sequences of different genes through specific amino acids in bHLH domains. The basic region of bHLH contributes to HRE recognition and the helix-loop-helix regions of theses domains form a helical bundle around the DNA and insert a major groove in a pseudo-symmetric fashion [22, 27]. Echinomycin, a natural cyclic peptide belonging to quinoxaline antibiotic family, has been introduced as a small inhibitor that interferes with the HIF-1–DNA interaction [19]. However, echinomycin is not the HIF specific inhibitor and confirmation of its cytotoxicity in several clinical trial phases has limited its clinical application [10].

More specifically, HIF-1a bHLH-PAS and Arnt bHLH-PAS truncated proteins have been reported to mediate the DNA-binding activity of HIF-1 [19]; however, no further examination has been done on these truncated proteins yet. In order to explore the HIF-1–DNA binding inhibition of proteins with small size, we designed a truncated protein, containing only the bHLH domains of HIF-1a and Arnt (which we called it ibHLH) to competent with HIF-1 in binding to the HRE sequence. To test our hypothesis, we induced hypoxia in HEK293T cells and transfected the cells with the PIRES2-EGFP vector expressing the ibHLH.

Firstly, hypoxia was successfully induced in HEK293T cells. CoCl_2_ treatment significantly increased gene expression levels of VEGF, vimentin and β-catenin and the protein level of HIF-1a. CoCl_2_ prevents HIF-1a hydroxylation by occupying an iron-binding site on the proline hydroxylases enzyme, thereby causing HIF-1a accumulation in normoxia condition [28]. The accumulated HIF-1a moves to the nucleus, dimers with Arnt and activates the expression of downstream genes of HIF-1, and is involved in angiogenesis, epithelial to mesenchymal transition (EMT), invasion, and metastasis [29–34].

Although CoCl_2_ increased the expression of the HIF-1a protein, HIF-1a mRNA was down regulated 48-h post treatment. Similar observations had been previously reported in breast cancer cell lines. Chu et al. showed that CoCl_2_ treatment increased the protein expression of HIF-1a in MCF-7 and MDA-MB-231 cell lines; however, mRNA expression decreased within the same periods of treatment, as we observed [35]. It seems that reduced levels of mRNA expression might be a negative feedback mechanism for inhibiting the HIF-1a accumulation following continuous exposure to CoCl_2_.

Secondly, the hypoxic cells were transfected with control and ibHLH expressing vectors. The ibHLH vector was successfully transcripted (Fig. 7a) and translated in the transfected cells (Fig. 4). However, further analysis indicated that ibHLH failed to significantly inhibit the activation of HIF-1 downstream genes, suggesting that some other regions of HIF-1 protein might be responsible for this process.

VEGF is the main downstream gene of HIF-1a and its overexpression has been used to validate hypoxia induction in most studies [36, 37]. In this study, overexpression of ibHLH has no effect on the gene expression of VEGF, while another HIF-DNA interfering molecule, echinomycin, has been reported to bind the HRE sequence in the VEGF promoter and directly inhibit the VEGF mRNA expression [19, 38]. Synthetic pyrrole-imidazole polyamide also targets the HRE sequence in the VEFG promoters, and inhibit the VEGF mRNA and protein level in vitro. However, their development as therapeutics remains to be further established [39]. Therefore, further investigation for developing a novel HIF-1 DNA binding inhibitor is still warranted.

Researches have demonstrated that low oxygen level is associated with the process of EMT through expression of HIF-1 protein [40]. HIF-1a increases the gene expression of snail and subsequently induces cell migration through increasing the mesenchymal markers such as N-cadherin and vimentin [41]. In contrast, β-catenin is directly regulated by HIF-1 through β-catenin-HRE interaction [42] or indirectly by expression of BCL9 in hypoxia [43]. Zhu et al. has reported HIF-1α siRNA significantly suppresses the expression of N-cadherin, both at the gene and the protein level, suggesting a possible therapeutic strategy to prevent hypoxia-induced EMT [44]. Recently, sanguinarine has been found to inhibit the EMT via targeting translocation of HIF-1α from cytosol to the nucleus during hypoxia [45]. However, to the best of our knowledge, the effect of DNA binding inhibitors such as echinomycin and polyamides on EMT markers have not been evaluated in the previous studies. In this study, we observed that overexpression of ibHLH mediated the hypoxia-dependent expression of β-catenin and N-cadherin. Although it was not statistically significant, it can be speculated that the affinity of ibHLH to HRE sequence was not strong enough to significantly inhibit the gene expression levels. Wu and his colleagues demonstrated the PAS domain of HIF-1a contributes to DNA binding affinity [22]. They showed that the PAS domain binds DNA at distance six base pairs away from the hexameric core element and mutations in PAS domain can reduce the affinity of HIF-1 to the HRE sequence. However, ibHLH had no effect in inhibiting the transcription of VEGF under hypoxia condition, as it is the main downstream gene of HIF. It seems that the function of PAS on DNA binding is not restricted to the affinity of DNA and may play a greater role in this process than previously suggested.

PAS domains were known as the main domains in dimerization of HIF-1a-Arnt. Targeting the PAS domain showed a promising result in inhibiting the dimerization of HIF-1a-Arnt and in further diminishing the HIF-1 transcriptional activity [46–49]. However, there are limited studies available about the capability of PAS in DNA interaction. For an appropriate HIF-1-HRE interaction, HIF-1 should recognize the HRE sequence, be able to bind this sequence and the affinity of interaction should be high. Current literature about the role of HIF domains in mentioned steps is limited and further studies regarding the specific role of PAS in DNA recognition, DNA binding and DNA affinity should be performed.

The research findings of this study were limited by lack of DNA-binding techniques such as quantitative Electrophoretic Mobility Shift Assay. This technique assesses the protein-DNA interaction [50] and is useful to study the attachment of ibHLH to HRE sequence and provides more robust evidence on this topic. Further studies with more specific DNA-binding assays are needed to reach a more conclusive result about the interaction of ibHLH and HRE sequence.

## Conclusion

In overall, despite of the critical role of bHLH in binding HIF-1 to HRE sequence of different genes, it seems that targeting this domain has failed to inhibit the endogenous transcriptional activity of HIF-1. Probably the presence of the PAS domain might increase the efficiency of this specific inhibitor. Further studies with more robust assays need to be performed to compare the effect of bHLH with bHLH-FAS in targeting the DNA-binding activity of HIF-1a pathway. Moreover, deciphering the molecular structure of HIF-1a domains and their critical functions in this pathway are important for developing a novel specific inhibitor.

## Supplementary information

**Additional file 1.** The sequencing result of the pIRES2-EGFP vector, containing the ibHLH DNA sequence.

## Data Availability

The datasets from the current study are included within the article.

## Abbreviations

HIF-1: Hypoxia inducible factor-1
HRE: Hypoxia response element
bHLH: Basic helix-loop-helix
ibHLH: Inhibitory bHLH
CoCl_2_: Cobalt chloride
ARNT: Aryl hydrocarbon receptor nuclear translocator
XRE: Xenobiotic response element
NMR: Nuclear magnetic resonance
PAS: Per-arnt-sim
DMEM: Dulbecco’s modified eagle medium
FBS: Fetal bovine serum
MTT: 3-(4,5-Dimethylthiazol-2-yl)-2,5-diphenyltetrazolium bromide
DMSO: Dimethyl sulfoxide
PBS: Phosphate buffered saline
BCA: Bicinchoninic acid
PVDF: Polyvinylidene difluoride
MCS: Multiple cloning site
PEI: Polyethylenimine
FDR: False discovery rate
EMT: Epithelial to mesenchymal transition
