# Supplementary figures and images for: Overexpression of bHLH domain of HIF-1 failed to inhibit the HIF-1 transcriptional activity in hypoxia
Source: Biol Res. 2020 Jun 5;53:25. doi: 10.1186/s40659-020-00293-4 (PMC7275393; doi:10.1186/s40659-020-00293-4)

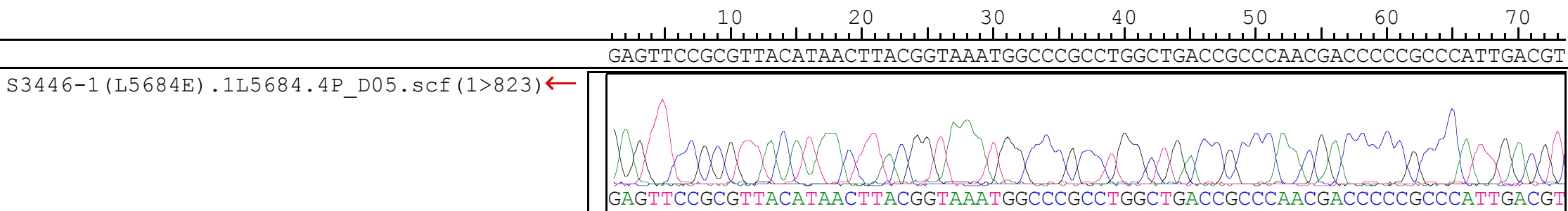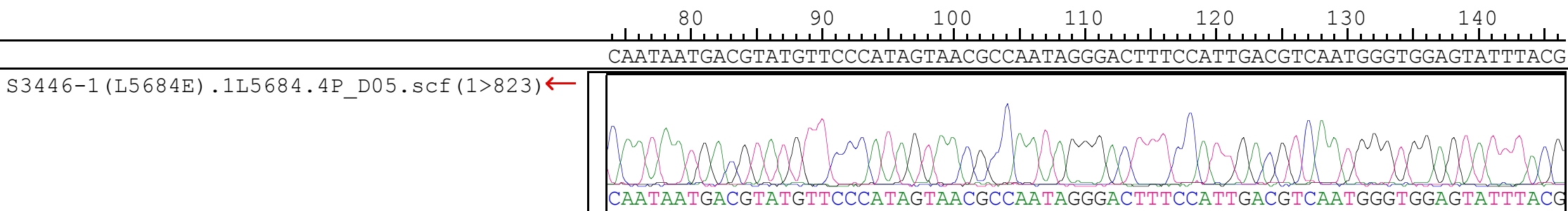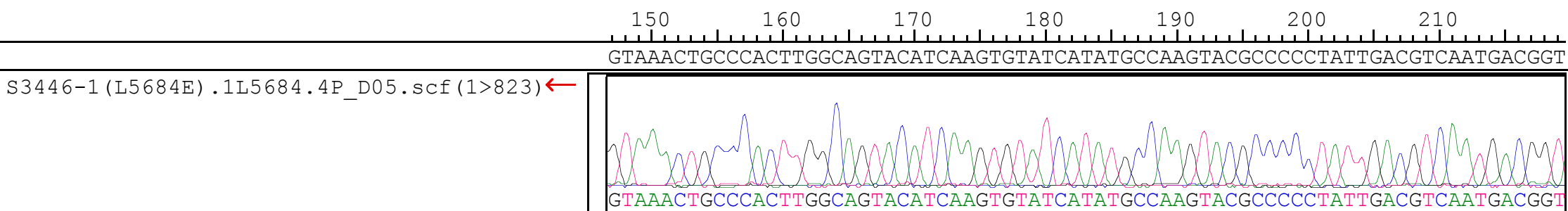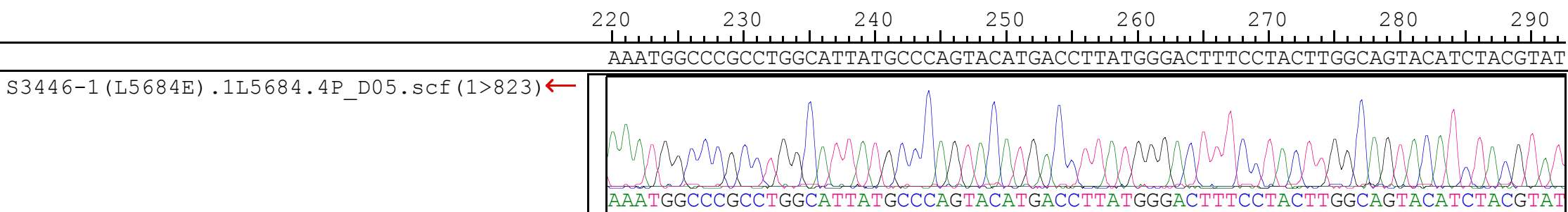

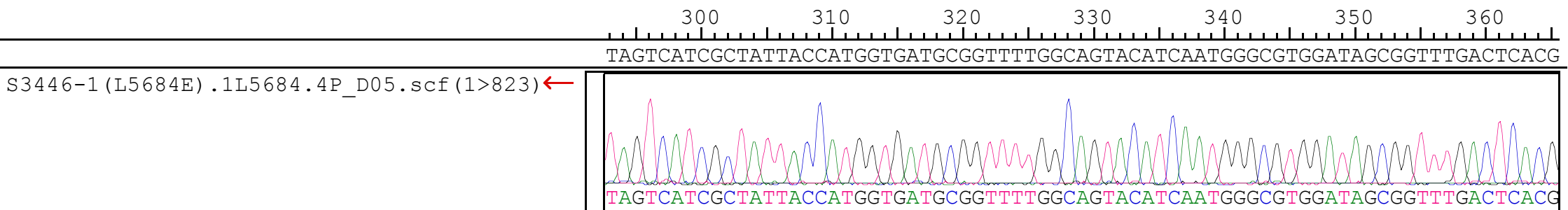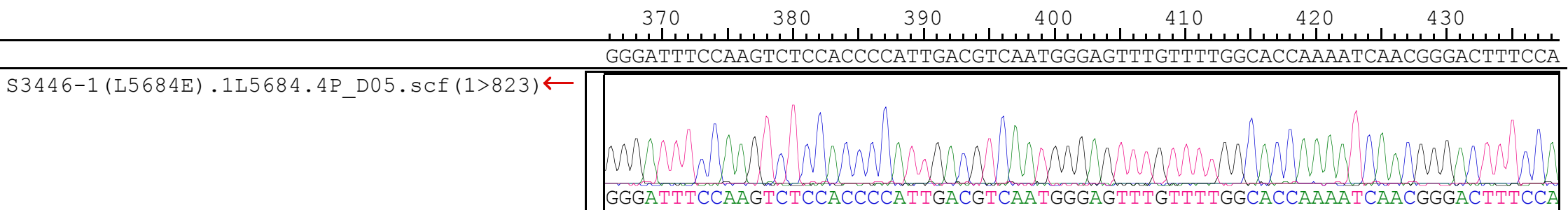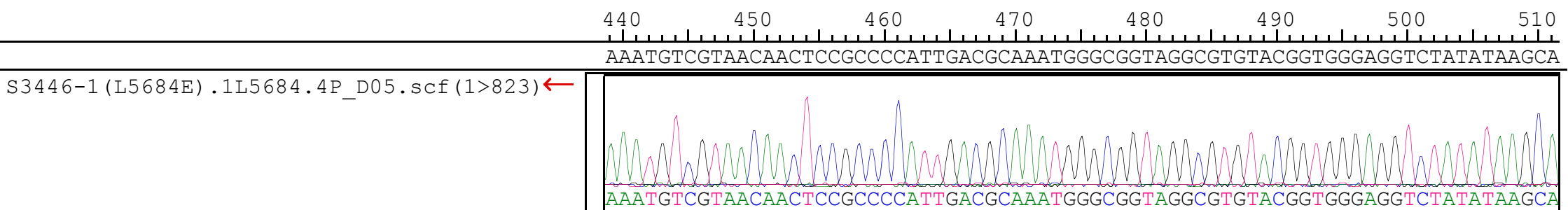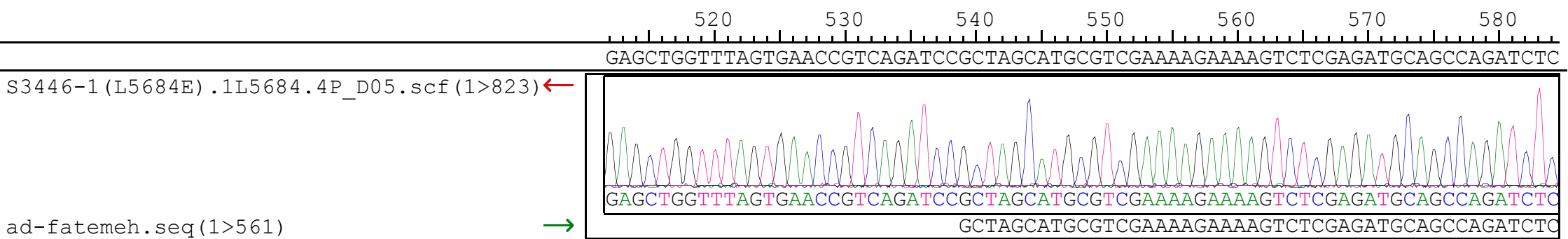

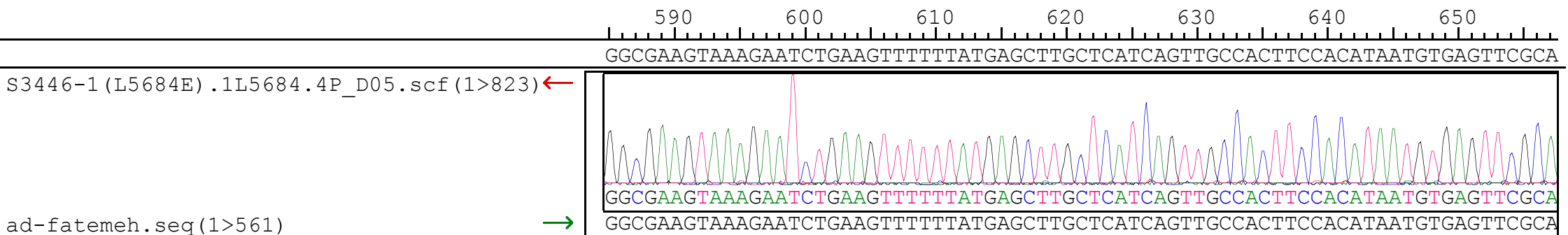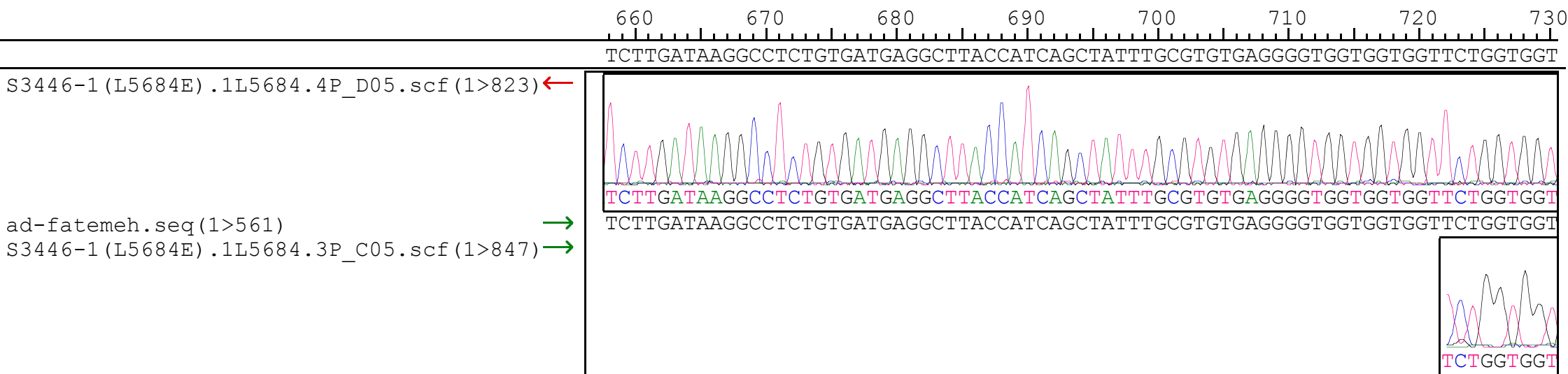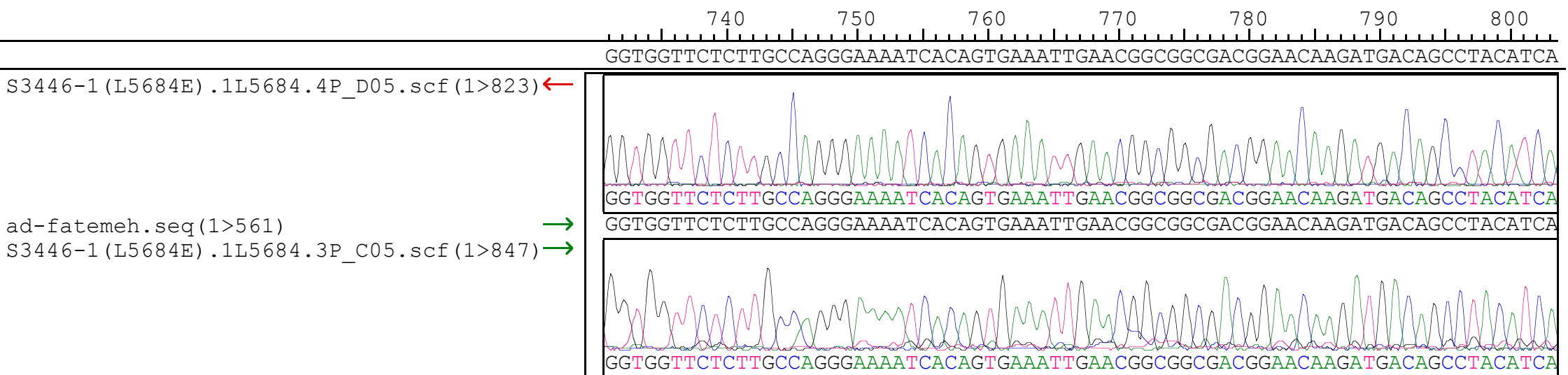

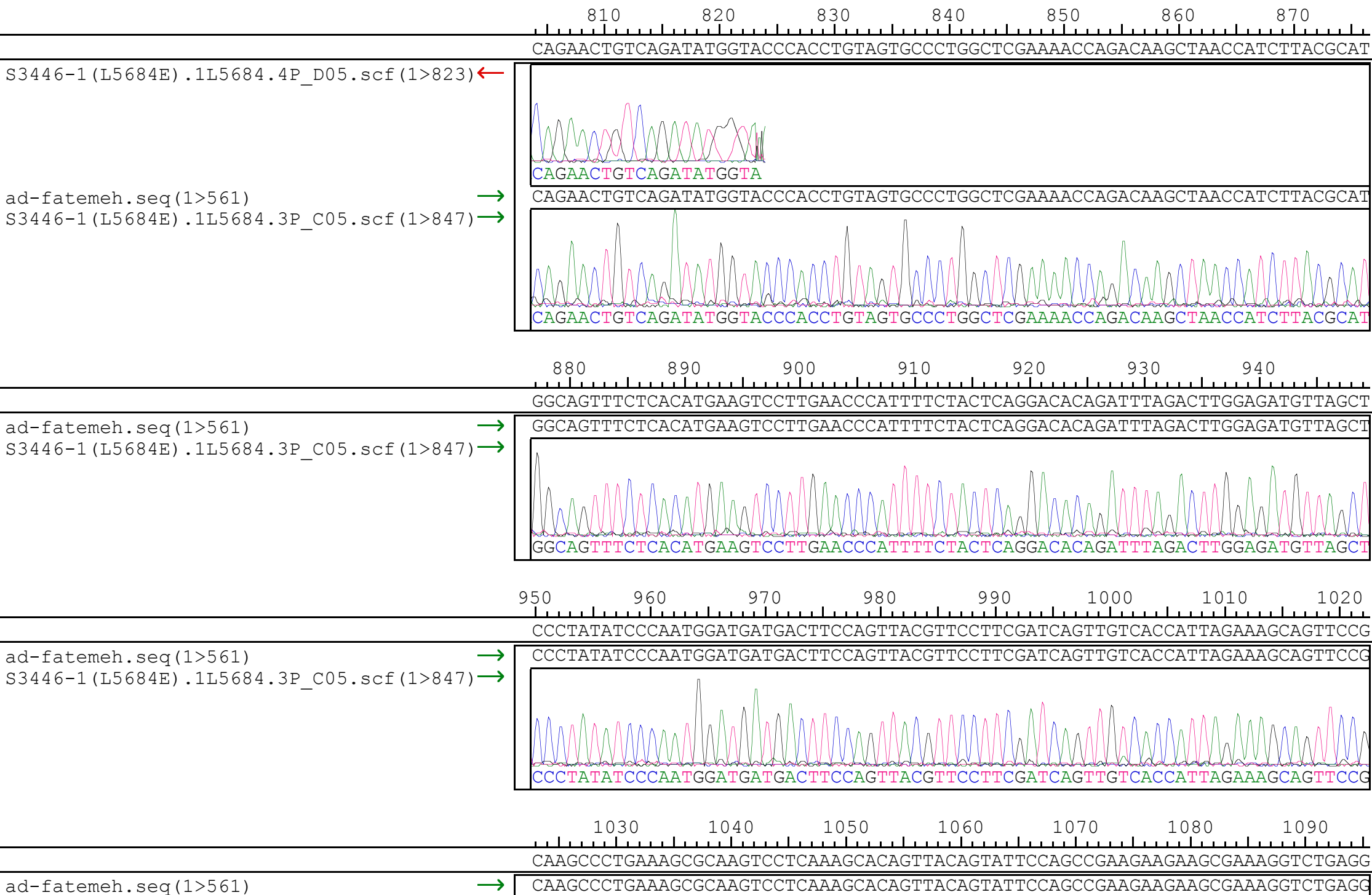

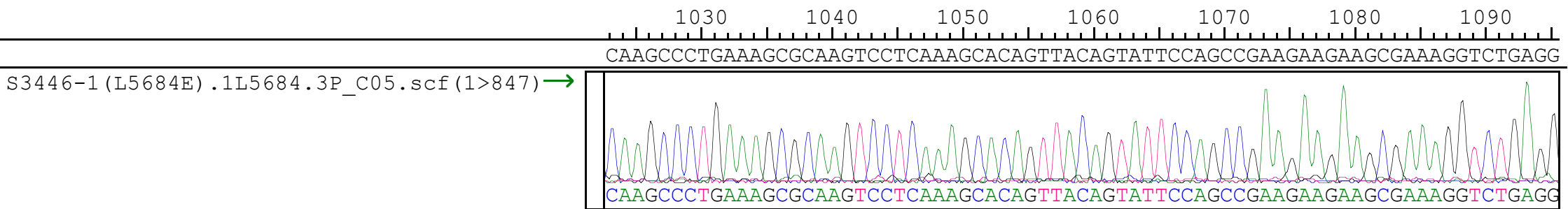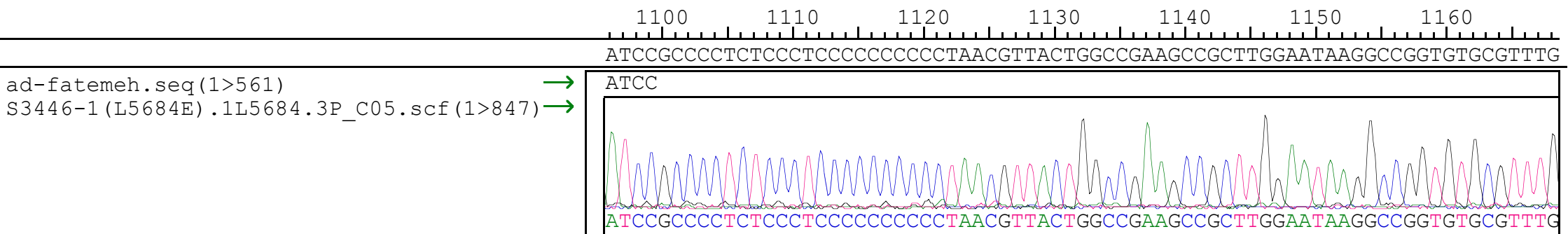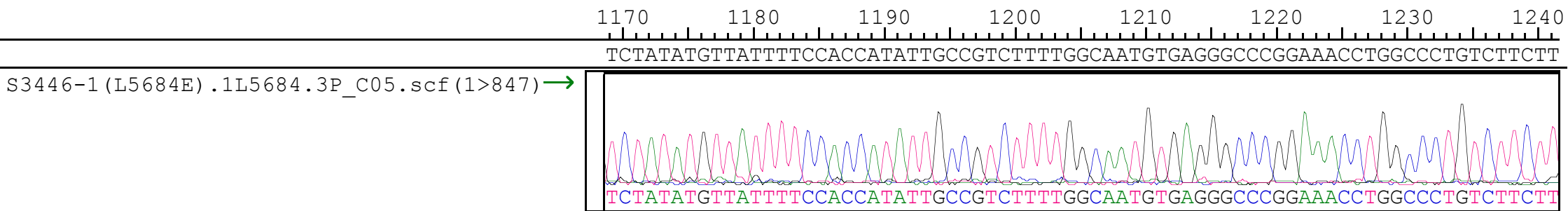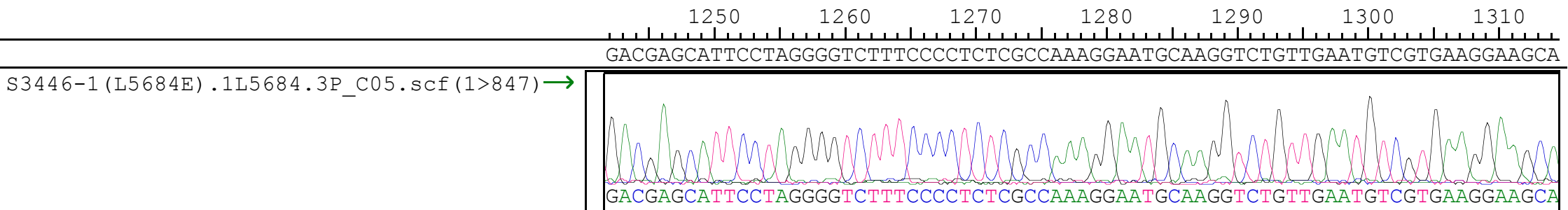

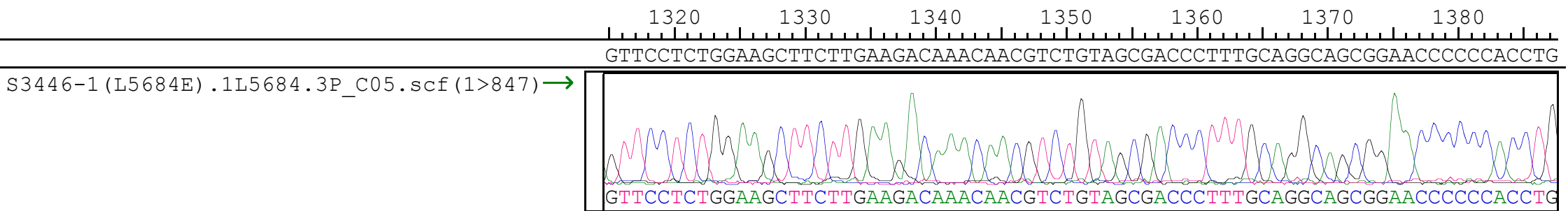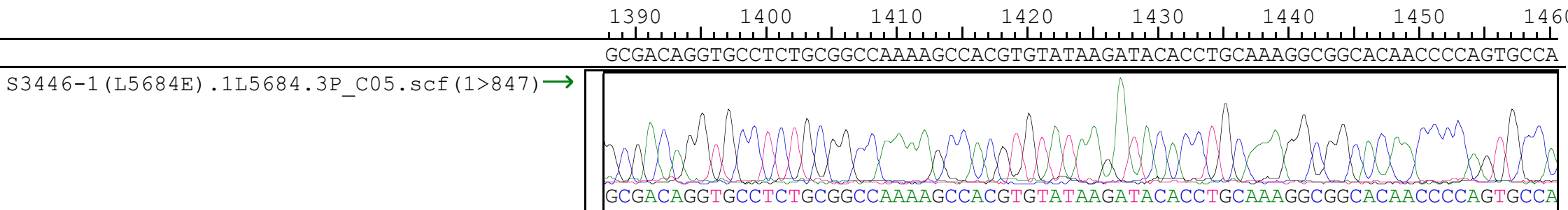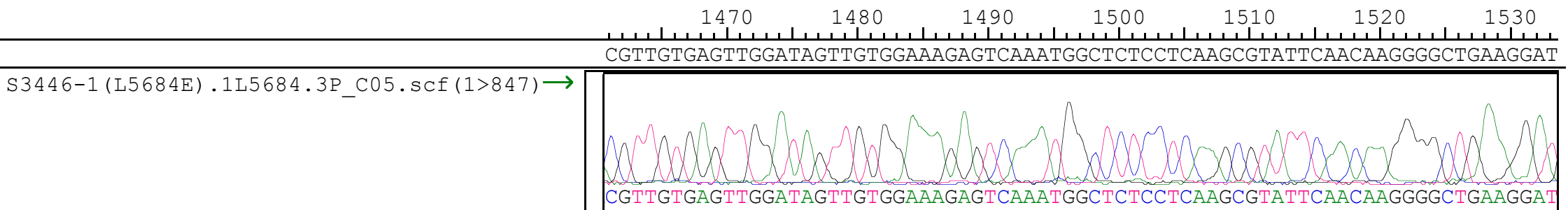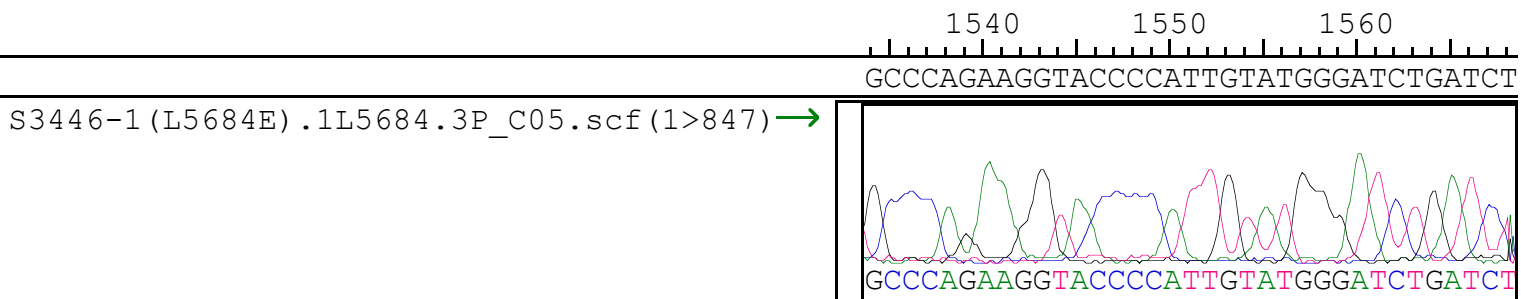

Supplement: Supplementary file 1 — Additional file 1. The sequencing result of the pIRES2-EGFP vector, containing the ibHLH DNA sequence. [file 40659_2020_293_MOESM1_ESM.pdf]
